# Supplementary figures and images for: Efficient depletion of ribosomal RNA for RNA sequencing in planarians
Source: BMC Genomics. 2019 Nov 29;20:909. doi: 10.1186/s12864-019-6292-y (PMC6884822; doi:10.1186/s12864-019-6292-y)

**A**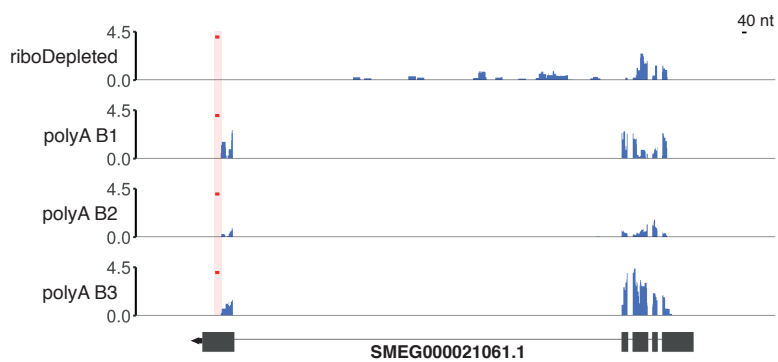**B**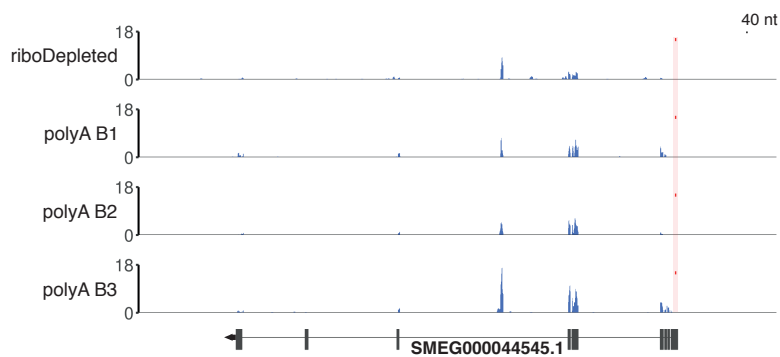**C**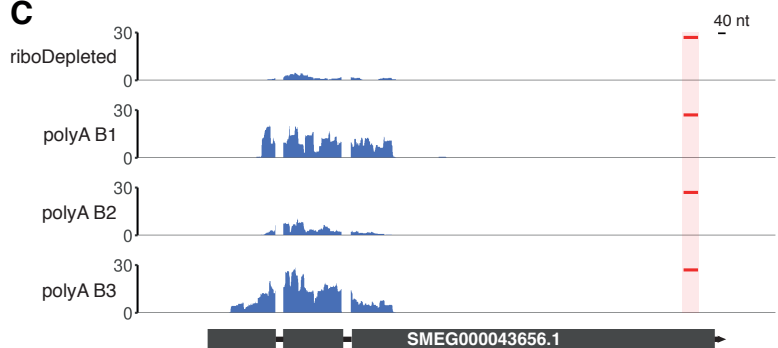**D**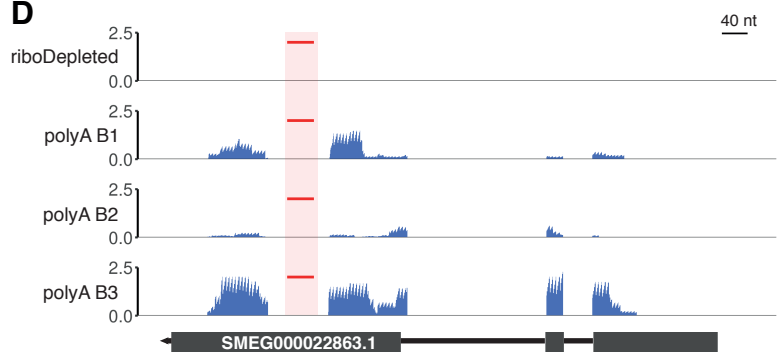

Supplement: Supplementary file 2 — Additional file 2: (A)-(D) RNA-seq coverage profile for genes potentially targeted by ribodepletion probes in rRNA depleted (ribodepleted) and poly(A) enriched (polyA B1, polyA B2, polyA B3) libraries. The position of antisense probes mapping to the transcripts is marked in red. [file 12864_2019_6292_MOESM2_ESM.pdf]
